# Plasma Metabolome and Metabolite Toxicity Profiling of Moderate-Intensity Running in Human Females

**DOI:** 10.3390/metabo16010043

**Published:** 2026-01-02

**Authors:** Qintong Fei, Tiantian Liang, Maodi Liang, Jing Cao, Huilin Yao, Ping Zhu, Qinghua Cui

**Affiliations:** 1 School of Sports Medicine, Wuhan Sports University, No. 461 Luoyu Rd., Hongshan District, Wuhan 430079, China; 2024410821@whsu.edu.cn (Q.F.); 2024410849@whsu.edu.cn (T.L.); liangmd633@163.com (M.L.); 2024420036@whsu.edu.cn (J.C.); 2024410824@whsu.edu.cn (H.Y.); 2 Department of Physical Education, Shanghai University of Traditional Chinese Medicine, 1200 Cailun Rd., Pudong New Area, Shanghai 201203, China; 3 Department of Biomedical Informatics, State Key Laboratory of Vascular Homeostasis and Remodeling, School of Basic Medical Sciences, Peking University, 38 Xueyuan Rd., Beijing 100191, China

**Keywords:** plasma metabolome, metabolic toxicity, algorithm, aerobic exercise

## Abstract

**Background**: Existing exercise metabolomics studies have predominantly focused on changes in the type and abundance of metabolites, while rarely addressing the toxicity risk of differential metabolites. Metabolic toxicity refers to the potential of endogenous or exogenous metabolites to induce oxidative stress, cell death, and other forms of biological damage when excessively accumulated and serves as a key driver of metabolic disorders. This study aims to characterize the toxicity risk of plasma differential metabolites before and after a single session of moderate-intensity running, so as to investigate the exercise-induced changes in metabolic toxicity. **Methods**: A single-group self-pretest–posttest control design was adopted in this study. Participants were recruited from Wuhan Sports University, China, with the inclusion criteria of healthy females aged 22–30 years and BMI 18.5–24.9. Individuals with a history of metabolic diseases or who met other exclusion criteria were excluded, and 5 females were finally enrolled. The exercise protocol consisted of a single 40 min session of moderate-intensity running on a treadmill. We collected plasma samples from five healthy females before and after exercise and performed untargeted LC-MS/MS metabolomic profiling. The gap-Δenergy algorithm was applied to calculate the toxicity scores of differential metabolites, and the proportion of metabolites with high toxic potential (score > 0.6) was compared. **Results**: Plasma metabolic profiles underwent notable remodeling after exercise. Thirty-two metabolites were upregulated and the phosphosphingolipid SM(d18:2(4E,14Z)/16:0) was the most significant. Meanwhile 32 metabolites were downregulated and the phosphosphingolipid PC(18:1(9Z)/14:0) was the most significant. The 64 differential metabolites were enriched in 9 KEGG pathways including amino acid metabolism and lipid metabolism. Moreover, we systematically evaluated the toxicity of these metabolites using the gap-Δenergy algorithm and found that the downregulated metabolites exhibited a significantly higher toxicity score compared to the upregulated ones. In addition, 37.5% of the downregulated metabolites had a high toxicity score, while the proportion of high toxicity in the upregulated group was only 15.6%. **Conclusions**: This study demonstrates that moderate-intensity running may confer metabolic health benefits to individuals by reducing metabolic toxicity, specifically through the downregulation of metabolites with high toxic potential. These findings offer novel evidence for exercise’s role in improving metabolic health. They also open a new direction for exercise-based interventions in metabolic disease–toxicity regulation.

## 1. Introduction

Exercise can promote extensive metabolic changes and molecular remodeling in skeletal muscles [1,2]. In addition, it can also regulate the activity of hepatic metabolic enzymes, improve renal excretory function, and modulate the hemodynamics of the cardiovascular system [3,4,5], with multiple organs and systems synergistically participating in the maintenance of metabolic homeostasis in the body, effectively alleviating various aspects of metabolic dysfunction in humans. As a result, it has been widely applied in the prevention, treatment, and management of metabolic diseases such as obesity and type 2 diabetes [6,7]. However, underlying mechanisms remain incompletely understood—particularly how exercise improves metabolic health by regulating metabolite homeostasis, which requires further in-depth analysis at the molecular level. The metabolome refers to all small-molecule metabolites in an organism [8], and the body’s response and adaptation to physiological stressors (e.g., exercise) can induce dynamic alterations in the metabolome [9]. Among these, the plasma metabolome serves as a key resource for uncovering the health benefits of exercise, as it can comprehensively and systematically reflect the body’s overall metabolic state [10,11].

Metabolic toxicity refers to the intrinsic toxic potential of metabolites and is recognized as one of the key drivers of metabolic dysfunction [12,13]. The excessive accumulation of endogenous or exogenous metabolites (e.g., certain amino acid derivatives and environmental pollutants) in the body may induce toxic effects such as oxidative stress [14,15], thereby activating downstream inflammatory pathways (e.g., the NF-κB signaling pathway) [16]. This oxidative stress-inflammation vicious cycle disrupts cellular homeostasis, impairs the integrity of metabolic pathways, and ultimately contributes to metabolic dysfunction [17,18]. Therefore, investigating exercise-induced changes in the toxicity risk of plasma metabolites is of great significance for elucidating the overall mechanism by which exercise improves metabolic health. However, current exercise metabolomics studies focus on exercise-induced changes in metabolite composition and abundance [19,20,21], but have overlooked the analysis of the toxicity of these differential metabolites—whether the exercise-induced changes in metabolites are accompanied by the selective regulation of toxicity risk. This gap limits our understanding of how exercise systematically maintains metabolic homeostasis from a toxicological perspective and hinders the identification of precision targets for exercise interventions.

Notably, acute moderate-intensity exercise is a commonly used model for investigating acute metabolic effects, as it can rapidly induce metabolome remodeling (e.g., energy substrate switching and metabolic waste clearance) [19,22]. Compared with long-term exercise interventions, this model avoids confounding factors arising from cumulative adaptive changes and can directly and accurately characterize the immediate metabolic responses induced by exercise. In addition, metabolome remodeling triggered by acute exercise serves as the basis for long-term exercise adaptation [1], and the findings derived therefrom can provide a theoretical basis for subsequent investigations into the impact of chronic exercise on metabolic toxicity. Thus, this model is of great value for exploring the selective regulatory effect of exercise on metabolic toxicity.

In this context, the present study aims to elucidate the acute effects of moderate-intensity exercise on plasma metabolome remodeling in healthy females and explore the selective regulatory patterns of exercise on the toxic potential of metabolites. We performed a metabolomic analysis of moderate-intensity running. Plasma samples were collected from 5 young healthy women before and after a single session of moderate-intensity running. Non-targeted LC-MS/MS metabolomics was employed for analysis, and the gap-Δenergy algorithm proposed by Zhang et al. [23] was used to calculate the toxicity score of differential metabolites. This study not only provides new insights into how exercise improves health via metabolic regulation, but also establishes a theoretical foundation for further exploring biomarkers from a toxicological perspective.

## 2. Materials and Methods

### 2.1. Study Participants and Ethics Approval

This study recruited 5 healthy female participants, aged between 22 and 30 years, with a body mass index (BMI) of 18.5–24.9 kg/m^2^. All subjects had no history of metabolic diseases, no history of sports injury or medication use in the past 3 months, and no regular exercise habits in the past 6 months (moderate-intensity exercise ≤ 1 time a week). We used the International Physical Activity Questionnaire (IPAQ) Short Form [24] to assess the participants’ physical activity levels. The total weekly physical activity volume of the participants was 528.5 ± 65.3 MET-min/week (mean ± SD), indicating they all belonged to the “low physical activity level” category. Additionally, the 1 min heart rate recovery test [25] was used to briefly evaluate cardiorespiratory endurance, with a 1 min heart rate recovery value of 18.3 ± 4.5 beats/minute, indicating good cardiorespiratory function.

This study was approved by the Medical Ethics Committee of Wuhan Sports University (approval number: 2025089), and all subjects signed a written informed consent before participating in the experiment. This study is an exploratory investigation adopting a small sample size to establish the methodological basis for innovative research, a widely accepted strategy in exercise metabolomics [26]. Furthermore, a small sample size can capture the preliminary trends in the toxic traits of metabolites, which serves as a theoretical foundation for subsequent large-scale studies. The rationale for selecting a single female population is to control for gender-related confounding factors, thereby more accurately capturing exercise-induced metabolic changes and ensuring the reliability of the study findings.

### 2.2. Exercise Intervention Protocol

The present study used the “acute moderate-intensity aerobic running” exercise model. The target heart rate (HR) range for each participant was calculated using the Karvonen formula: target HR = (220 − age − resting HR) × exercise intensity + resting HR [27]. The exercise intensity was set to 40%—60%, corresponding to an average target running HR of 123–147 bpm. Resting heart rate of each participant was measured using a Polar H10 HR band (Polar Electro, Kempele, Finland) on the morning of 3 days before exercise: after fasting and resting quietly for 10 min, heart rate was continuously recorded for 1 min, and the average value over the 3 days was taken as the individual resting heart rate. The experiment was conducted at the National Fitness Center of Wuhan Sports University during the participants’ non-menstrual period.

To control for confounding factors, the subjects followed a standardized diet (55% carbohydrates, 20% protein, and 25% fat of total energy) [28], and alcohol and caffeine intake were prohibited during this period. The experimental environment was maintained at a room temperature of 24 ± 2 °C and a relative humidity of 50 ± 5%. During exercise, participants first warmed up on a treadmill at 4.0 km/h for 5 min, then ran at their target HR for 30 min, and finally cooled down at 4.0 km/h for 5 min to stabilize their HR. HR was monitored in real time throughout the process using a Polar H10 HR band.

### 2.3. Plasma Sample Collection and Processing

Plasma samples were collected at the Sports Medicine Laboratory of Wuhan Sports University under at 25 ± 3 °C and 49 ± 6% relative humidity. Pre-exercise blood collection was conducted between 8:30 and 9:00 AM, while post-exercise blood collection was completed within 10 min after the termination of exercise (10:00–11:00 AM), with a time window deviation of ≤20 min for all samples. Systolic blood pressure (SBP), diastolic blood pressure (DBP), and resting heart rate of the participants were measured during a 30 min rest period prior to exercise.

Before exercise (following 30 min of rest) and within 15 min after exercise, 5 mL of blood was collected from the cubital vein of each subject. During blood collection, all participants maintained a standardized sitting posture (upper arm kept at the same level as the heart), and blood samples were collected by certified medical personnel in accordance with clinical venous blood collection operating standards. Within 30 min of blood collection, the samples were centrifuged at 1800× *g* for 5 min and then stored at 4 °C. Subsequently, the supernatant (plasma) was aspirated, aliquoted, and stored in a minus 80 °C refrigerator until analysis.

### 2.4. Untargeted Metabolomic Sequencing

Frozen plasma samples were thawed at 4 °C and vortexed for homogenization; a 100 μL aliquot of plasma was transferred to an EP tube, and 300 μL of pre-chilled pure methanol was added before the mixture was incubated at −20 °C for 30 min. The mixture was then centrifuged at 16,000× *g* and 4 °C for 20 min, and the supernatant was collected for mass spectrometry (MS) analysis. This study used an ultra-high performance liquid chromatography-tandem mass spectrometry (UPLC-MS/MS) system for detection, including a Shimadzu Nexera X2 LC-30AD UPLC system (Shimadzu, Kyoto, Japan), an Orbitrap Fusion MS instrument (Thermo Scientific, Waltham, MA, USA), and a Waters ACQUITY UPLC^®^ HSS T3 chromatographic column (2.1 × 100 mm, 1.8 μm, Waters, Milford, MA, USA).

For chromatographic separation, samples were placed in a 4 °C autosampler with an injection volume of 16 μL, the column temperature was maintained at 40 °C, and the flow rate was set to 0.3 mL/min; the chromatographic mobile phase A was 0.1% formic acid in water, and mobile phase B was 0.1% formic acid in acetonitrile. The chromatographic gradient elution program was as follows: 0–2 min at 0%B, 2–3.3 min with B linearly increasing to 48%, 3.3–5.1 min with B linearly increasing to 100%, 5.1–7.2 min at 100%B, 7.2–7.3 min with B linearly decreasing to 0%, and 7.3–10 min at 0%B. In terms of mass spectrometry data acquisition, samples were detected in both positive (+) and negative (−) ion modes using electrospray ionization (ESI), with the following ionization conditions: Spray Voltage of 3.8 kV (+) and 3.2 kV (−), Capillary Temperature of 320 °C (for both modes), Sheath Gas of 40 (arbitrary units, for both modes), Aux Gas of 15 (arbitrary units, for both modes), Probe Heater Temp of 350 °C (for both modes), and S-Lens RF Level of 50.

Equal volumes of all plasma samples to be tested were mixed to prepare quality control (QC) samples; by comparing the base peak chromatograms (BPC) of QC samples in positive and negative ion modes, the results showed good consistency in the response intensity and retention time of all chromatographic peaks, indicating reliable instrument detection stability and minimal variation caused by instrument error.

### 2.5. Identifying Differential Metabolites

MSDIAL 4.9 [29] was used for peak alignment, retention time correction and peak area extraction of original mass spectrometry data; metabolite structure identification was achieved by retrieving the human metabolite database (HMDB), MassBank mass spectrometry database, and Global Natural Products Social Molecular Networking (GNPS) through accurate mass matching (mass deviation < 10 ppm) and MS/MS spectrum matching (mass deviation < 0.01 Da). The data filtering criteria were as follows: ion peaks with >50% missing values within groups were deleted, data from positive and negative ion modes were normalized by total peak area separately and then integrated, Python 3.10 software was used for pattern recognition, and the data were subjected to unit variance scaling (UV) before being used for subsequent analysis.

R 4.5.0 was used for statistical analysis and visualization, with univariate and multivariate analyses applied to identify significant differential metabolites. Paired t-tests (before vs. after exercise) were used to calculate the *p*-value of each metabolite, with the screening criteria set as *p* < 0.05 and fold change (FC) > 4/3 or <3/4. Principal component analysis (PCA) score plot and multidimensional scaling (MDS) plot were generated, and a partial least squares discriminant analysis (PLS-DA) model was constructed; the validity of the model was verified by 200 permutation tests to avoid overfitting, and important metabolites were screened using the variable importance in projection (VIP) threshold (VIP > 1) to finally determine the differential metabolites. Hierarchical clustering analysis and Spearman’s correlation analysis were also performed on the top 30 differential metabolites ranked by VIP value.

### 2.6. Calculation of Toxicity Scores for Differential Metabolites

The toxicity score of differential metabolites was calculated using Python 3.13, based on the gap-Δenergy algorithm [23]. Using the SMILES (Simple Molecular-Input Line-Entry System) of metabolites (obtained from the HMDB database), the molecular feature gap-Δenergy was calculated by constructing a molecular graph with chemical bonds as nodes, calculating the gap and Δenergy values of node pairs, and aggregating these values into features such as mean and standard deviation; combined with traditional molecular descriptors, a machine learning model incorporating XGBoost was built, and a stacking ensemble strategy was used to optimize prediction performance. The toxicity score was normalized to range from 0 to 1, with a value closer to 1 indicating higher confidence that the model predicts the metabolite as a toxic molecule. The Wilcoxon rank-sum test was used to compare the toxicity risk of upregulated and downregulated differential metabolites after exercise, and Fisher’s exact test was used to compare the proportion of metabolites with high toxicity score (>0.6) between the two groups.

### 2.7. Metabolic Pathway Enrichment Analysis

The MetaboAnalystR 5.0 package [30] was used for pathway enrichment analysis of differential metabolites based on the Kyoto Encyclopedia of Genes and Genomes (KEGG) database [31], with the threshold for significant enrichment set to *p* < 0.05. Topological pathway impact was calculated based on relative betweenness centrality, and the pathway activity DA score was calculated as follows: DA score = (number of metabolites significantly increased after exercise − number of metabolites significantly decreased after exercise)/total number of detected metabolites in the pathway. The DA score ranges from −1 to 1, with a positive value indicating an overall increase in metabolite levels in the pathway and a negative value indicating an overall decrease.

## 3. Results

### 3.1. Baseline Characteristics of the Study Participants

**Figure 1:**
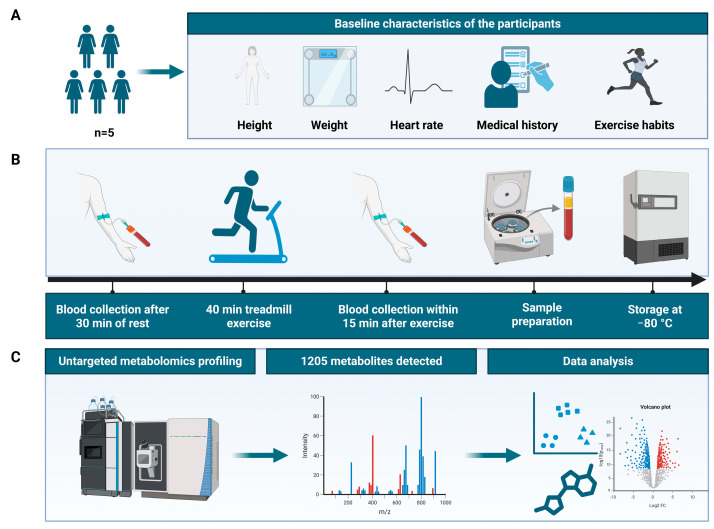
The workflow of this study. (**A**) Participant recruitment and baseline assessment; (**B**) exercise intervention, sample collection, and processing; (**C**) untargeted metabolomic analysis.

The overall workflow of this study is shown in Figure 1. According to the preset inclusion and exclusion criteria (no history of metabolic diseases, no regular exercise history in the past 6 months, BMI of 18.5–24.9 kg/m^2^, etc.), five healthy female participants were finally enrolled in the study. The baseline characteristics of the participants were as follows (mean ± SD): age, 25 ± 1.87 years; height, 1.63 ± 0.05 m; BMI, 19.85 ± 1.92 kg/m^2^; body fat percentage, 24.4 ± 1.73%; and resting heart rate (HR), 75.60 ± 7.44 beats per minute. During the exercise intervention, the participants maintained an actual HR of 132.5 ± 8.7 beats per minute, and the proportion of exercise duration within the target HR range (123–147 bpm) relative to the total exercise time (40 min) was 92.5 ± 5.3%, meeting the preset criteria for moderate-intensity exercise. Individual baseline data of each participant are provided in Supplementary Table S1.

### 3.2. Characteristics of the Overall Plasma Metabolomic Profile

**Figure 2:**
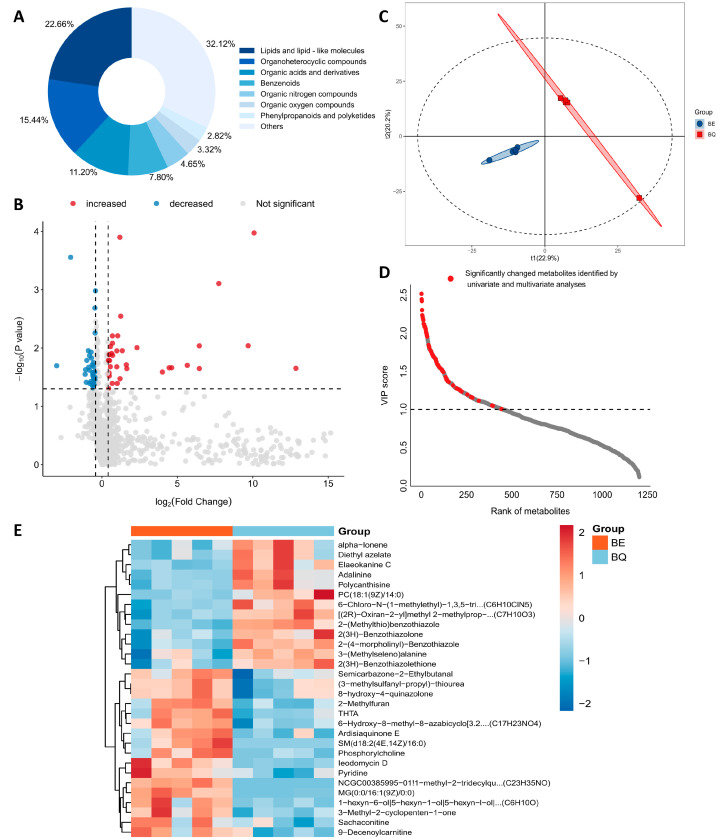
Plasma metabolomic characteristics before and after exercise. (**A**) Pie chart of plasma metabolite classification based on the Super Class of HMDB; (**B**) Volcano plot of differential metabolites. Screening thresholds: paired t-test *p* < 0.05 and fold change (FC) < 0.75 (3/4) or >1.33 (4/3); red dots represent significantly upregulated metabolites, blue dots represent significantly downregulated metabolites, and gray dots represent metabolites with no significant difference; (**C**) PLS-DA score plot of pre-exercise (BQ) and post-exercise (BE) samples; (**D**) VIP score plot of the PLS-DA model. Screening threshold for differential metabolites: VIP > 1; red dots represent metabolites with significant changes after exercise; (**E**) Heatmap of expression patterns of the top 30 differential metabolites ranked by VIP value. Red indicates increased relative expression of metabolites, blue indicates decreased expression, and the color gradient reflects the degree of change in expression levels.

After untargeted UPLC-MS/MS metabolomic sequencing and data filtering, 1205 stable metabolites were finally retained for subsequent analysis (Supplementary Table S2). According to the Super Class classification of the Human Metabolome Database (HMDB), these metabolites were categorized into 8 classes (Figure 2A), including lipids and lipid-like molecules, organoheterocyclic compounds, organic acids and derivatives, benzenoids, organic nitrogen compounds, organic oxygen compounds, phenylpropanoids and polyketides, and others (accounting for <2% of total metabolites and unclassified metabolites).

Principal Component Analysis (PCA) was used to assess the overall metabolic variation in the metabolic profile and data quality. Pre-exercise and post-exercise samples showed a clear separation trend along PC1 and PC2, which accounted for 33.47% and 19.36% of the total variance of the dataset, respectively (Supplementary Figure S1A). Furthermore, we employed multidimensional scaling (MDS) to visualize the sample metabolomic profiles, using 1—Spearman’s correlation coefficient as the distance metric (Supplementary Figure S1B). The results showed that pre-and post-exercise samples exhibited distinct clustering separation along MDS dimension 1 and dimension 2, further verifying the significant difference in metabolomic profiles between the two groups and suggesting that exercise remodeled the plasma metabolome.

### 3.3. Post-Exercise-Altered Plasma Metabolome

To characterize the post-exercise-altered plasma metabolome, we analyzed differential metabolites and their interaction networks. The volcano plot (Figure 2B) visualizes the significant differences in metabolites between the two groups (pre-exercise vs. post-exercise) via univariate analysis. For multivariate analysis, we performed partial least squares discriminant analysis (PLS-DA) to further verify the inter-group separation (Figure 2C) and identified 451 metabolites that contributed notably to inter-group variation (Figure 2D). Two hundred permutation tests were conducted to evaluate the validity of the discriminant model and avoid overfitting (Supplementary Figure S2).

Through cross-validation of univariate and multivariate analyses, 64 differential metabolites were identified (Supplementary Table S3), including 16 organoheterocyclic compounds, 13 lipids and lipid-like molecules, and 10 organic acids and derivatives. Among these, 32 metabolites were upregulated and 32 were downregulated. The most significantly downregulated metabolite was phosphatidylcholine PC(18:1(9Z)/14:0) (log_2_FC = −3.00, *p* = 0.02, VIP = 2.17), while the most markedly elevated one was sphingomyelin SM(d18:2(4E,14Z)/16:0) (log_2_FC = 12.86, *p* = 0.02, VIP = 2.09), suggesting that exercise exerts directional regulatory effects on phospholipid and sphingolipid metabolism. The downregulation of phosphatidylcholine may be associated with cell membrane remodeling during exercise [32], and the upregulation of sphingomyelin may be involved in regulating post-exercise signal transduction and cell proliferation [33,34].

**Figure 3:**
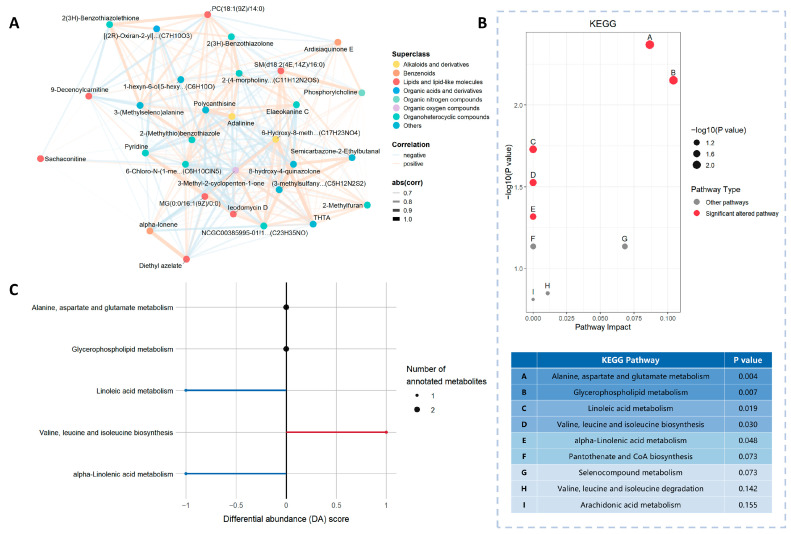
Regulatory characteristics of exercise on metabolic pathways. (**A**) Correlation network diagram of differential metabolites. Nodes represent the top 30 differential metabolites ranked by VIP, analyzed using Spearman’s correlation analysis (after FDR correction, *p* < 0.05, absolute correlation coefficient |corr| > 0.6). Node color represents metabolite class (Superclass); edges’ color (red/blue) and thickness correspond to the direction of correlation (positive/negative) and the magnitude of the correlation coefficient (|corr| value), respectively; (**B**) KEGG pathway enrichment bubble plot. Red dots represent pathways with *p* < 0.05; (**C**) DA score plot of metabolic pathway activity. The differential abundance (DA) score reflects the overall regulatory trend of exercise on different metabolic pathways, ranging from −1 to 1 (−1 indicates an overall decrease in metabolite levels in the pathway, while 1 indicates an overall increase). The size of the dots represents the number of annotated metabolites in the pathway.

A heatmap was constructed for the top 30 differential metabolites ranked by VIP using hierarchical clustering based on Euclidean distance, which clearly distinguished the pre-exercise and post-exercise groups (Figure 2E). The metabolite correlation network diagram (Figure 3A) shows the relationships with notable Spearman’s correlation coefficients (FDR < 0.05, |corr| > 0.6) (Supplementary Table S4). The topological structure of this network indicates that metabolites interact closely, with the densest interactions observed between lipids/lipid-like molecules and organic oxygen compounds—suggesting these are core modules of exercise-induced metabolic regulation. The extensive interactions between these metabolites may mediate processes such as energy metabolism and oxidative stress responses during exercise [35], acting as a key regulatory node for maintaining metabolic balance during exercise. Overall, these findings show that a highly coordinated metabolite regulatory network is the basis of exercise-induced metabolic remodeling.

### 3.4. Exercise Regulates Multiple Metabolic Pathways

In the pathway analysis, the 64 differential metabolites were enriched in 9 KEGG pathways. Among them, we identified 5 notably changed pathways, involving amino acid metabolism and lipid metabolism (Figure 3B and Supplementary Table S5). The differential abundance (DA) scores of these five pathways are shown in Figure 3C: the activity of valine, leucine, and isoleucine biosynthetic pathways was enhanced, while two lipid metabolism pathways—linoleic acid metabolism and alpha-linolenic acid metabolism—were notably downregulated. In addition, in the alanine, aspartate and glutamate metabolism pathway, N-Acetyl-L-aspartic acid (log_2_FC = 0.48, *p* = 0.01) is significantly upregulated, while 2-Oxosuccinamate (log_2_FC = −0.55, *p* = 0.03) exhibited a significant downward trend. Although the DA score of this pathway was 0, this reverse regulation of metabolites reflects a precise metabolic response to exercise. In summary, exercise exerts a multi-dimensional regulatory effect on amino acid and lipid metabolism, which provides further evidence for the molecular mechanism by which exercise improves metabolic health.

### 3.5. Toxicity Risk of Downregulated Metabolites Is Higher than That of Upregulated Ones

**Figure 4:**
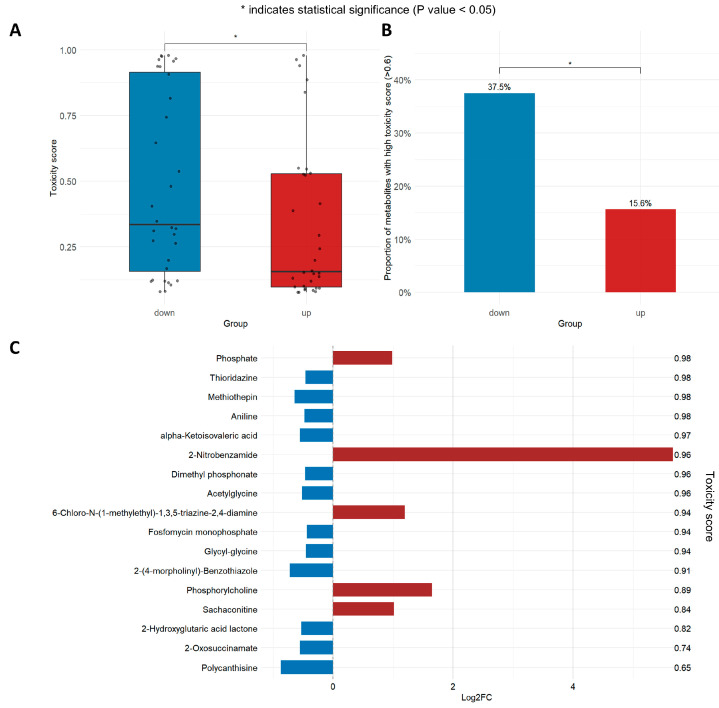
Toxicity score change of differential metabolites after exercise. (**A**) Box plot of toxicity score for downregulated and upregulated differential metabolites. The box represents the interquartile range (IQR), the horizontal line inside the box denotes the median, and scatter points represent the toxicity score value of each individual metabolite; (**B**) Bar chart of the proportion of metabolites with high toxicity score (>0.6); (**C**) Bidirectional bar chart of log_2_FC and toxicity score for metabolites with high toxicity score. Blue bars represent metabolites downregulated after exercise, and red bars represent those upregulated after exercise; the length of the bars corresponds to log_2_FC, and the values on the right side indicate the toxicity score value of the corresponding metabolite.

For the 64 exercise-related differential metabolites, we calculated their toxicity score using the gap-Δenergy algorithm [23], combined with the metabolites’ SMILES structures; details are provided in Supplementary Table S6. Interestingly, the average toxicity score of downregulated metabolites was 0.49 ± 0.35, which was markedly higher than that of upregulated metabolites (0.33 ± 0.30; W = 659, *p* < 0.05) (Figure 4A). Among metabolites in the downregulated group, those with high toxicity risk (score > 0.6) made up 37.5%, compared to only 15.6% in the upregulated group, showing a meaningful difference in distribution between the two groups (*p* < 0.05) (Figure 4B). The 12 downregulated metabolites with high toxicity potential included 5 metabolites related to amino acid metabolism pathways (e.g., alpha-ketoisovaleric acid, acetylglycine), 4 xenobiotic metabolites (e.g., thioridazine), 2 metabolites related to organophosphorus metabolism pathways (e.g., dimethyl phosphonate), and 1 alkaloid metabolite (polycanthisine) (Figure 4C).

The results show that exercise may improve metabolic health by lowering the risk of toxic metabolites and reducing the accumulation of potentially toxic metabolites in the body. Toxic metabolites refer to those with a toxicity score > 0.6 calculated using the gap-Δenergy algorithm. This score reflects the potential toxicity risk of metabolites rather than validated biological toxicity. It is worth noting that among the elevated metabolites with a high toxicity score, the increase in the aromatic nitro xenobiotic compound 2-nitrobenzamide is the most significant. This indicates that the downregulatory effect of exercise on such compounds may be weaker than that on other types of xenobiotics. Notably, the findings are based on small-sample exploratory analyses. Further large-sample cohort studies are therefore required to validate the regulatory patterns of metabolites with high toxic potential and their causal associations with metabolic health.

## 4. Discussion

This study found that the plasma metabolome was significantly remodeled after exercise. Enrichment analysis showed that differential metabolites were mainly distributed in pathways such as amino acid metabolism and lipid metabolism. Among these, the biosynthetic pathways of branched-chain amino acids (BCAAs; e.g., the biosynthesis pathways of valine, leucine, and isoleucine) were upregulated, which may be closely related to the body’ s energy requirements during exercise. When muscle glycogen was depleted, BCAAs can supplement blood glucose via gluconeogenesis or activate the mTOR pathway to promote muscle protein synthesis, thereby facilitating post-exercise recovery [36,37,38]. This result was consistent with the findings of Morville et al. [22], who analyzed their blood samples from 10 healthy males after endurance exercise (EE). Their results showed that EE also activated BCAAs synthesis. A meta-analysis by Khemtong et al. [39] also indicated that BCAAs supplementation can alleviate muscle damage and improve muscle soreness in males after exercise.

Importantly, we found that downregulated metabolites exhibited a markedly higher average toxicity score than their upregulated counterparts after exercise, and the proportion of metabolites with high toxicity score (>0.6) was also higher in the downregulated group. The decreased metabolites with high toxicity risk included both endogenous metabolic intermediates (e.g., alpha-ketoisovaleric acid) and xenobiotic metabolites (e.g., thioridazine). Taking alpha-ketoisovaleric acid (log_2_FC = −0.55, *p* = 0.02, toxicity probability = 0.97) as an example: it is a key intermediate in valine catabolism, and its excessive accumulation can interfere with hepatic ketone body synthesis (increasing the risk of ketoacidosis) and inhibit BCAAs uptake by skeletal muscle, thereby exacerbating post-exercise muscle catabolism [40,41]. The downregulation of alpha-ketoisovaleric acid after exercise may synergistically improve glucose and lipid metabolic homeostasis by preserving BCAAs for muscle protein synthesis and reducing excessive ketone body production [37,42].

In summary, based on the results of this study, we hypothesize that exercise may synergistically downregulate metabolites with a high toxicity risk through multiple pathways. First, exercise may upregulate the activity of liver drug-metabolizing enzymes, such as the cytochrome P450 enzyme system and glutathione S-transferases (GSTs) [43,44]. Existing studies support the mechanism that aerobic exercise can enhance the activity of hepatic detoxifying enzymes, which will further enhance the oxidation and conjugation reactions of endogenous toxic intermediates and exogenous toxic metabolites, and accelerate their degradation and inactivation [45,46]. Second, moderate-intensity exercise can significantly increase the glomerular filtration rate (GFR), as confirmed in clinical studies on exercise-induced renal hemodynamic changes, thereby promoting the filtration and excretion of toxic metabolites, and reducing their toxic load in vivo [47]. Finally, physiological oxidative stress may induced by exercise may activate pathways such as Nrf2, induce the synthesis of endogenous antioxidant molecules (e.g., glutathione), and enhance the clearance capacity of metabolites with potential oxidative damage (e.g., some alkaloids), which further consolidates the regulatory network underlying exercise-mediated reduction in metabolic toxicity [48,49]. This is a reasonable speculation based on the oxidative stress pathway. However, further in vitro or in vivo experiments are required to verify its direct regulatory role in metabolic toxicity clearance.

Existing studies mostly focus on the effect of exercise on metabolite abundance [50,51]. For instance, Koay et al. [11] found that after 80 days of aerobic exercise in healthy males, many lipid metabolic intermediates—such as malonate (FC = −9.1, *p* = 4.7 × 10^−13^) and arachidonate (ARA) (FC = −1.39, *p* = 6.6 × 10^−8^)—were notably decreased. In addition, existing studies also largely focus on the effect of exercise on exogenous toxicants [52,53]. For example, one study reported that exercise can reduce the impairment of cardiovascular, autonomic nervous, and immune functions caused by the organophosphorus compound diisopropyl fluorophosphate (DFP) in mice [52]. In contrast, this study is the first to link exercise to metabolite toxicity using a toxicity risk model. This provides a new perspective for exercise to improve metabolic health: exercise not only alters the abundance of metabolites, but also gives priority to reducing the level of plasma metabolites with highly toxic potential, so as to reduce cell and tissue damage caused by metabolic toxicity [54,55]. Beyond revealing a new molecular mechanism by which exercise reduces the risk of metabolic diseases, this finding further provides a new target for exercise-based intervention in metabolic diseases.

However, there are still several limitations in the current study. First, this study obtained high-dimensional data using untargeted metabolomics technology, with a sample size of only 5, leading to a risk of false-positive results. Second, the toxicity scores derived from the gap-Δenergy algorithm in this study are model-predicted outcomes, which only reflect the potential toxicity risk of metabolites rather than indicating that metabolites exert definite toxicity to the human body; subsequent in vitro cell assays or similar experiments are required to verify their actual biological toxic effects. Third, in this study, a formula was employed to estimate the participants’ maximal heart rate (HRmax) for determining the target heart rate zone of exercise. However, individual differences were not fully taken into account, which may give rise to slight deviations. In future research, it is advisable to incorporate evaluation indicators such as measured VO_2_max. In addition, only young female participants were recruited in the current study, while this approach reduces gender-related confounding factors, it also limits the generalizability of the findings. Meanwhile, besides healthy people, it is also necessary to investigate those with specific pathological conditions (e.g., diabetes). It would be interesting to explore the abnormal characteristics of core metabolic pathways under disease states, as well as the regulatory effects of exercise interventions on these pathways and the toxic potential of related metabolites. This can provide a more precise theoretical basis for exercise therapy of metabolic diseases. Moreover, this study only focuses on a single session of moderate-intensity running; it is quite important to explore other types of exercise. Finally, the duration of changes in metabolite toxicity risk and their association with long-term health still need to be explored. Therefore, future research can expand the sample size to verify the generalizability of the results, identify the optimal exercise protocol for regulating metabolite toxicity through multiple exercise intervention groups, clarify the regulatory differences in metabolite toxic potential among different exercise types and intensities, to provide a more precise basis for personalized exercise interventions, and analyze the association between changes in metabolite toxicity and the risk of metabolic diseases. This will further explore the feasibility of using high toxicity risk metabolites as markers for evaluating the efficacy of exercise interventions.

## 5. Conclusions

In summary, by analyzing plasma samples collected from five healthy women before and after a single 40 min moderate-intensity run, this study is the first to systematically evaluate the toxic risk of the differential metabolites post-exercise, which suggests that moderate-intensity running prioritizes the downregulation of metabolites with high toxic potential. However, as this study is a small-sample exploratory investigation, it only provides preliminary evidence for potential associations. In the field of exercise science, this finding expands our understanding of the metabolic health benefits of exercise to the dimension of toxicity regulation, facilitating the exploration of toxicity-related biomarkers. Furthermore, for the prevention and treatment of metabolic diseases, the present study proposes a novel potential mechanism: exercise may improve metabolic homeostasis by reducing the accumulation of metabolites with toxic potential, thereby alleviating cell and tissue damage induced by metabolic toxicity. This provides a new target for exercise intervention strategies, such as optimizing exercise protocols to enhance the clearance of metabolites with toxic potential.

## Data Availability

All data supporting the findings of this study are included in the article and Supplementary Materials and can be obtained from the corresponding author upon request. Source data are provided with this article.

## Supplementary Materials

The following supporting information can be downloaded at: https://www.mdpi.com/article/10.3390/metabo16010043/s1, Supplementary Figure S1. Metabolomic separation between pre-exercise and post-exercise samples; Supplementary Figure S2. The permutation test of partial least squares discriminant analysis (PLS-DA) model; Supplementary Table S1. Participant Characteristics; Supplementary Table S2. Total Plasma Metabolites; Supplementary Table S3. Differential Metabolites; Supplementary Table S4. Spearman Correlation; Supplementary Table S5. Enriched Pathway; Supplementary Table S6. Toxicity Score.

## References

[1] Egan B., Zierath J.R. (2013). Exercise metabolism and the molecular regulation of skeletal muscle adaptation. Cell Metab..

[2] Hargreaves M., Spriet L.L. (2020). Skeletal muscle energy metabolism during exercise. Nat. Metab..

[3] Farzanegi P., Dana A., Ebrahimpoor Z., Asadi M., Azarbayjani M.A. (2019). Mechanisms of beneficial effects of exercise training on non-alcoholic fatty liver disease (NAFLD): Roles of oxidative stress and inflammation. Eur. J. Sport Sci..

[4] Poortmans J.R., Vanderstraeten J. (1994). Kidney function during exercise in healthy and diseased humans. An update. Sports Med..

[5] Duncker D.J., Bache R.J. (2008). Regulation of coronary blood flow during exercise. Physiol. Rev..

[6] Yang Z., Scott C.A., Mao C., Tang J., Farmer A.J. (2014). Resistance exercise versus aerobic exercise for type 2 diabetes: A systematic review and meta-analysis. Sports Med..

[7] O’Donoghue G., Blake C., Cunningham C., Lennon O., Perrotta C. (2021). What exercise prescription is optimal to improve body composition and cardiorespiratory fitness in adults living with obesity? A network meta-analysis. Obes. Rev..

[8] Wishart D.S. (2019). Metabolomics for Investigating Physiological and Pathophysiological Processes. Physiol. Rev..

[9] Kelly R.S., Kelly M.P., Kelly P. (2020). Metabolomics, physical activity, exercise and health: A review of the current evidence. Biochim. Biophys. Acta Mol. Basis Dis..

[10] He Y., Zhang Y., Lai J., Ma S., Yin P., Wu Z., Zhou J. (2025). Plasma metabolomics dataset of race-walking athletes illuminating systemic metabolic reaction of exercise. Sci. Data.

[11] Koay Y.C., Stanton K., Kienzle V., Li M., Yang J., Celermajer D.S., O’sUllivan J.F. (2021). Effect of chronic exercise in healthy young male adults: A metabolomic analysis. Cardiovasc. Res..

[12] Le Daré B., Lagente V., Gicquel T. (2019). Ethanol and its metabolites: Update on toxicity, benefits, and focus on immunomodulatory effects. Drug Metab. Rev..

[13] Zhao Q., Wu Z.E., Li B., Li F. (2022). Recent advances in metabolism and toxicity of tyrosine kinase inhibitors. Pharmacol. Ther..

[14] Durand P., Prost M., Loreau N., Lussier-Cacan S., Blache D. (2001). Impaired homocysteine metabolism and atherothrombotic disease. Lab. Investig..

[15] Peillex C., Pelletier M. (2020). The impact and toxicity of glyphosate and glyphosate-based herbicides on health and immunity. J. Immunotoxicol..

[16] Reuter S., Gupta S.C., Chaturvedi M.M., Aggarwal B.B. (2010). Oxidative stress, inflammation, and cancer: How are they linked?. Free Radic. Biol. Med..

[17] Deretic V. (2021). Autophagy in inflammation, infection, and immunometabolism. Immunity.

[18] Lee Y.S., Olefsky J. (2021). Chronic tissue inflammation and metabolic disease. Genes Dev..

[19] Li K., Schön M., Naviaux J.C., Monk J.M., Alchus-Laiferová N., Wang L., Straka I., Matejička P., Valkovič P., Ukropec J. (2022). Cerebrospinal fluid and plasma metabolomics of acute endurance exercise. FASEB J..

[20] Dünnwald T., Paglia G., Weiss G., Denti V., Faulhaber M., Schobersberger W., Wackerhage H. (2022). High Intensity Concentric-Eccentric Exercise Under Hypoxia Changes the Blood Metabolome of Trained Athletes. Front. Physiol..

[21] Mueller-Hennessen M., Sigl J., Fuhrmann J.C., Witt H., Reszka R., Schmitz O., Kastler J., Fischer J.J., Müller O.J., Giannitsis E. (2017). Metabolic profiles in heart failure due to non-ischemic cardiomyopathy at rest and under exercise. ESC Heart Fail..

[22] Morville T., Sahl R.E., Moritz T., Helge J.W., Clemmensen C. (2020). Plasma Metabolome Profiling of Resistance Exercise and Endurance Exercise in Humans. Cell Rep..

[23] Zhang S., Zhao D., Cui Q. (2024). Gap-Δenergy, a New Metric of the Bond Energy State, Assisting to Predict Molecular Toxicity. ACS Omega.

[24] Lee P.H., Macfarlane D.J., Lam T.H., Stewart S.M. (2011). Validity of the International Physical Activity Questionnaire Short Form (IPAQ-SF): A systematic review. Int. J. Behav. Nutr. Phys. Act..

[25] Cole C.R., Blackstone E.H., Pashkow F.J., Snader C.E., Lauer M.S. (1999). Heart-rate recovery immediately after exercise as a predictor of mortality. N. Engl. J. Med..

[26] Dong G., Liu H., Chen Y., Bao D., Xu W., Zhou J. (2024). Hydrogen-Rich Gas Enhanced Sprint-Interval Performance: Metabolomic Insights into Underlying Mechanisms. Nutrients.

[27] Karvonen J., Vuorimaa T. (1988). Heart rate and exercise intensity during sports activities. Practical application. Sports Med..

[28] Kerksick C., Harvey T., Stout J., Campbell B., Wilborn C., Kreider R., Kalman D., Ziegenfuss T., Lopez H., Landis J. (2008). International Society of Sports Nutrition position stand: Nutrient timing. J. Int. Soc. Sports Nutr..

[29] Tsugawa H., Cajka T., Kind T., Ma Y., Higgins B., Ikeda K., Kanazawa M., VanderGheynst J., Fiehn O., Arita M. (2015). MS-DIAL: Data-independent MS/MS deconvolution for comprehensive metabolome analysis. Nat. Methods.

[30] Chong J., Xia J. (2018). MetaboAnalystR: An R package for flexible and reproducible analysis of metabolomics data. Bioinformatics.

[31] Kanehisa M., Goto S. (2000). KEGG: Kyoto encyclopedia of genes and genomes. Nucleic Acids Res..

[32] Piccarducci R., Daniele S., Fusi J., Chico L., Baldacci F., Siciliano G., Bonuccelli U., Franzoni F., Martini C. (2019). Impact of ApoE Polymorphism and Physical Activity on Plasma Antioxidant Capability and Erythrocyte Membranes. Antioxidants.

[33] Górski J., Dobrzyn A., Zendzian-Piotrowska M. (2002). The sphingomyelin-signaling pathway in skeletal muscles and its role in regulation of glucose uptake. Ann. N. Y. Acad. Sci..

[34] Carpio L., Stephan E., Kamer A., Dziak R. (1999). Sphingolipids stimulate cell growth via MAP kinase activation in osteoblastic cells. Prostaglandins Leukot. Essent. Fat. Acids.

[35] Gibb A.A., Hill B.G. (2018). Metabolic Coordination of Physiological and Pathological Cardiac Remodeling. Circ. Res..

[36] Lynch C.J., Adams S.H. (2014). Branched-chain amino acids in metabolic signalling and insulin resistance. Nat. Rev. Endocrinol..

[37] Gaggini M., Carli F., Bugianesi E., Gastaldelli A., Rosso C., Buzzigoli E., Marietti M., Della Latta V., Ciociaro D., Abate M.L. (2018). Altered amino acid concentrations in NAFLD: Impact of obesity and insulin resistance. Hepatology.

[38] Xu M., Hu D., Liu X., Li Z., Lu L. (2025). Branched-Chain Amino Acids and Inflammation Management in Endurance Sports: Molecular Mechanisms and Practical Implications. Nutrients.

[39] Khemtong C., Kuo C.-H., Chen C.-Y., Jaime S.J., Condello G. (2021). Does Branched-Chain Amino Acids (BCAAs) Supplementation Attenuate Muscle Damage Markers and Soreness after Resistance Exercise in Trained Males? A Meta-Analysis of Randomized Controlled Trials. Nutrients.

[40] Xu Y., Jiang H., Li L., Chen F., Liu Y., Zhou M., Wang J., Jiang J., Li X., Fan X. (2020). Branched-Chain Amino Acid Catabolism Promotes Thrombosis Risk by Enhancing Tropomodulin-3 Propionylation in Platelets. Circulation.

[41] Mitsubuchi H., Owada M., Endo F. (2005). Markers associated with inborn errors of metabolism of branched-chain amino acids and their relevance to upper levels of intake in healthy people: An implication from clinical and molecular investigations on maple syrup urine disease. J. Nutr..

[42] Puchalska P., Crawford P.A. (2017). Multi-dimensional Roles of Ketone Bodies in Fuel Metabolism, Signaling, and Therapeutics. Cell Metab..

[43] Gollasch B., Dogan I., Rothe M., Gollasch M., Luft F.C. (2019). Maximal exercise and plasma cytochrome P450 and lipoxygenase mediators: A lipidomics study. Physiol. Rep..

[44] Sen C.K., Marin E., Kretzschmar M., Hanninen O. (1992). Skeletal muscle and liver glutathione homeostasis in response to training, exercise, and immobilization. J. Appl. Physiol..

[45] Guengerich F.P. (2019). Cytochrome P450 research and The Journal of Biological Chemistry. J. Biol. Chem..

[46] Vaish S., Gupta D., Mehrotra R., Mehrotra S., Basantani M.K. (2020). Glutathione S-transferase: A versatile protein family. 3 Biotech.

[47] Traise A., Dieberg G., Pearson M.J., Smart N.A. (2024). The effect of exercise training in people with pre-dialysis chronic kidney disease: A systematic review with meta-analysis. J. Nephrol..

[48] Kretzschmar M., Müller D. (1993). Aging, training and exercise. A review of effects on plasma glutathione and lipid peroxides. Sports Med..

[49] Wang P., Li C.G., Qi Z., Cui D., Ding S. (2016). Acute exercise stress promotes Ref1/Nrf2 signalling and increases mitochondrial antioxidant activity in skeletal muscle. Exp. Physiol..

[50] Khoramipour K., Sandbakk Ø., Keshteli A.H., Gaeini A.A., Wishart D.S., Chamari K. (2022). Metabolomics in Exercise and Sports: A Systematic Review. Sports Med..

[51] Contrepois K., Wu S., Moneghetti K.J., Hornburg D., Ahadi S., Tsai M.-S., Metwally A.A., Wei E., Lee-McMullen B., Quijada J.V. (2020). Molecular Choreography of Acute Exercise. Cell.

[52] Freire Machi J., Schmidt R., Salgueiro L.M., Stoyell-Conti F.F., Barboza C.d.A., Hernandez D.R., Morris M. (2019). Exercise benefits the cardiac, autonomic and inflammatory responses to organophosphate toxicity. Toxicol. Rep..

[53] Zhang X., Cao L., Ji B., Li L., Qi Z., Ding S. (2020). Endurance training but not high-intensity interval training reduces liver carcinogenesis in mice with hepatocellular carcinogen diethylnitrosamine. Exp. Gerontol..

[54] Deng P., Li X., Petriello M.C., Wang C., Morris A.J., Hennig B. (2019). Application of metabolomics to characterize environmental pollutant toxicity and disease risks. Rev. Environ. Health.

[55] Kong L., Zhao Q., Jiang X., Hu J., Jiang Q., Sheng L., Peng X., Wang S., Chen Y., Wan Y. (2024). Trimethylamine N-oxide impairs β-cell function and glucose tolerance. Nat. Commun..

