# Supplementary material for: Plasma Metabolome and Metabolite Toxicity Profiling of Moderate-Intensity Running in Human Females
Source: Metabolites. 2026 Jan 2;16(1):43. doi: 10.3390/metabo16010043 (PMC12844069; doi:10.3390/metabo16010043)

Supplementary Figure S1. Metabolomic separation between pre-exercise and post-exercise samples. (A) The plot of PCA score across pre-exercise (BQ) and post-exercise samples (BE).
(B) Multidimensional scaling (MDS) plot with 1 - Spearman ’ s correlation coefficient as the distance metric, showing the distinct clustering of BE and BQ groups.

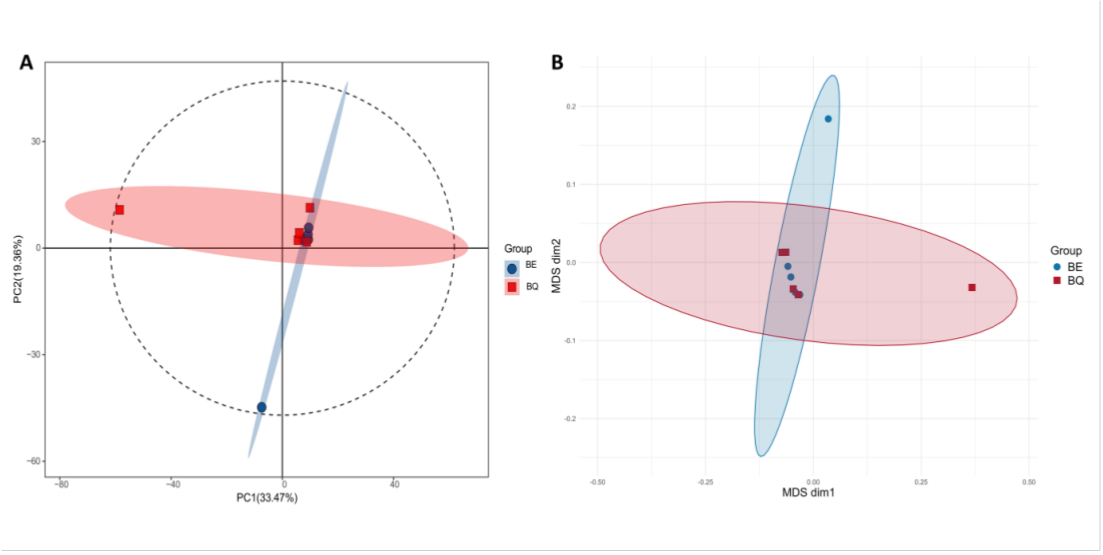

Supplementary Figure S2. The permutation test of partial least squares discriminant analysis (PLS-DA) model. R2Y represents the percentage of predictor variance explained by the full model. Q2 indicates the predictive performance of the model. The distribution characteristics show that the original R2Y and Q2 (at Similarity (Y, Yperm) = 1) are significantly higher than those from permutations, indicating that the PLS-DA model is not overfitted.

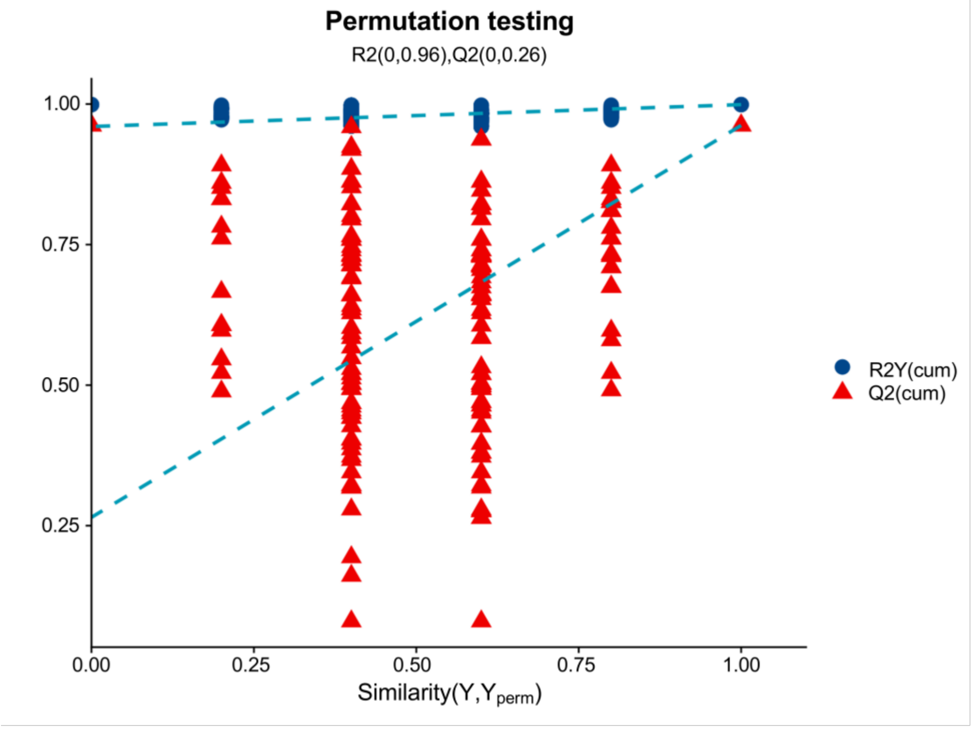

Supplement: Supplementary file 1 [file metabolites-16-00043-s001.zip › Supplementary Figure.pdf]
